# Supplementary material for: The impact of wearing facemask on COPD patients: A protocol of a systematic review and meta-analysis
Source: PLoS One. 2023 Sep 28;18(9):e0292388. doi: 10.1371/journal.pone.0292388 (PMC10538665; doi:10.1371/journal.pone.0292388)
Supplement: Attachment — Submitted filename: Response to Reviewers-V12.docx [file pone.0292388.s002.docx]

Dr Dong Keon Yon

Academic Editor

PLOS ONE

1 Aug 2023

Dear Dr Yon

We appreciate the constructive comments made by the reviewers, and our responses are as follows:

Comments to the Author

*1. Does the manuscript provide a valid rationale for the proposed study, with clearly identified and justified research questions?*

*The research question outlined is expected to address a valid academic problem or topic and contribute to the base of knowledge in the field.*

Reviewer #1: *Partly*

Reviewer #2: *Partly*

Reviewer #3: *Yes*

Reviewer #4: *No*

We have made corrections and edited our manuscript accordingly.

*2. Is the protocol technically sound and planned in a manner that will lead to a meaningful outcome and allow testing the stated hypotheses?*

*The manuscript should describe the methods in sufficient detail to prevent undisclosed flexibility in the experimental procedure or analysis pipeline, including sufficient outcome-neutral conditions (e.g. necessary controls, absence of floor or ceiling effects) to test the proposed hypotheses and a statistical power analysis where applicable. As there may be aspects of the methodology and analysis which can only be refined once the work is undertaken, authors should outline potential assumptions and explicitly describe what aspects of the proposed analyses, if any, are exploratory.*

Reviewer #1: *Partly*

Reviewer #2: *No*

Reviewer #3: *Partly*

Reviewer #4: *No*

We have made corrections and edited our manuscript accordingly.

3. *Is the methodology feasible and described in sufficient detail to allow the work to be replicable?*

*Descriptions of methods and materials in the protocol should be reported in sufficient detail for another researcher to reproduce all experiments and analyses. The protocol should describe the appropriate controls, sample size calculations, and replication needed to ensure that the data are robust and reproducible.*

Reviewer #1: *Yes*

Reviewer #2: *No*

Reviewer #3: *Yes*

Reviewer #4: *No*

We have made corrections and edited our manuscript accordingly.

4. *Have the authors described where all data underlying the findings will be made available when the study is complete?*

*The PLOS Data policy requires authors to make all data underlying the findings described in their manuscript fully available without restriction, with rare exception, at the time of publication. The data should be provided as part of the manuscript or its supporting information, or deposited to a public repository. For example, in addition to summary statistics, the data points behind means, medians and variance measures should be available. If there are restrictions on publicly sharing data—e.g. participant privacy or use of data from a third party—those must be specified.*

Reviewer #1: *No*

Reviewer #2: *Yes*

Reviewer #3: *No*

Reviewer #4: *No*

We have modified our manuscript in the “Data Availability Statement”. (Data Availability Statement, lines 304-306, paragraph 2 page 16)

*5. Is the manuscript presented in an intelligible fashion and written in standard English?*

*PLOS ONE does not copyedit accepted manuscripts, so the language in submitted articles must be clear, correct, and unambiguous. Any typographical or grammatical errors should be corrected at revision, so please note any specific errors here.*

Reviewer #1: *Yes*

Reviewer #2: *No*

Reviewer #3: *Yes*

Reviewer #4: *No*

Thanks. This manuscript has been proofread by a native English speaker with scientific background.

6. *Review Comments to the Author*

Reviewer #1: *The protocol seems to be interesting but, I have a few questions for the authors.*

*1. One group is the COPD patients who are wearing masks and the other group comprises of normal people/ COPD patients who are not wearing masks?*

During the study period, the trial group of COPD patients wore masks, while the other group served as the control and either did not wear facemasks or wore different facemasks. To clarify this point, we have modified our manuscript accordingly. It now reads: “We will include COPD patients who did not wear facemasks or wore different facemasks as controls, compared to the experimental group who wore masks during the study period.” (Methods, line 130-132 paragraph 2 page 7)

*2. Are you taking into account the vaccination history of these patients like influenza or the pneumococcal because they could have an impact on the number of exacerbations?*

*3. What about* *the medication history of the patients including oral and inhaled steroids?*

*4. The educational background of the individual and the caregiver support at home is also an important factor to be considered. Is it being done in this study?*

We believe that points 2, 3, and 4 are closely related, and our response is as follows:

To clarify this point, we have modified our manuscript to include the following: “1. Participant characteristics, including sex, age, disease severity, vaccination history, medication history, educational background, caregiver support at home, and other relevant factors.” (Methods, line 230-232 paragraph 2 page 12)

Reviewer #2: *The study design appears retrospective, looking at databases. Yet it implies intervention in terms of mask wear. Furthermore, it implies checking at EtCO2, 6MWT at end of study. Intervention and prospective assessment cannot be done in a retrospective study.*

We apologize for any confusion. To clarify, we have added the following sentences to the manuscript: “Although we will search the database for published outcomes, the experiment design we intend to include is a prospective study that comprises prospective intervention trials for both randomized and non-randomized controlled trials.” (Methods, line 115-118 paragraph 2 page 6)

Reviewer #3: *Authors are required to present the plan of data availability in respective question. Although data is not generated yet even then a data sharing plan may be given as accorded by the Plos One policy.*

We have modified our manuscript in the “Data Availability Statement”. (Data Availability Statement, lines 304-306, paragraph 2 page 16)

Reviewer #4: *This manuscript is a proposal of potential meta-analysis about the impact of wearing facemask on the clinical outcomes of COPD patients. I have below comments.*

*1. It’s not clear about the importance of this topic about the correlation between wearing facemask and the clinical outcomes of COPD patients. If the environment and air quality is good, facemask would be unnecessary for COPD patients.*

To clarify this point, we have added the following sentences to the manuscript: “According to the GOLD 2023 committee report, there is a supra-linear relationship between air pollution and respiratory events, indicating that there is no absolutely safe level of air pollution [17]. Wearing a facemask would benefit the general population, particularly COPD patients who are more vulnerable to invisible pollution and could experience acute exacerbations [18]. Therefore, it is advisable for COPD patients to wear masks in normal and/or specific environments to minimize disease progression [19].” (Introduction, line 88-94 paragraph 1 page 5)

17. Sin DD, Doiron D, Agusti A, Anzueto A, Barnes PJ, Celli BR, et al. Air pollution and COPD: GOLD 2023 committee report. Eur Respir J. 2023; 61(5):2202469. https://doi:10.1183/13993003.02469-2022 PMID: 36958741
18. Jiang XQ, Mei XD, Feng D. Air pollution and chronic airway diseases: what should people know and do? J Thorac Dis. 2016; 8(1):E31-40. https://doi:10.3978/j.issn.2072-1439.2015.11.50 PMID: 26904251
19. Kyung SY, Jeong SH. Particulate-Matter Related Respiratory Diseases. Tuberc Respir Dis (Seoul). 2020; 83(2):116-21. https://doi: 10.4046/trd.2019.0025 PMID: 32185911

*2. It is not clear how many published studies are available for meta-analysis to summarize the correlation between wearing facemask and the clinical outcomes of COPD patients. Authors should have provided such information to confirm the feasibility of the proposed systemic review.*

To clarify this point, we have added the following sentences to the manuscript: “This is a protocol for investigating the potential impact of wearing masks among COPD patients. To ensure feasibility, this protocol plans to search without limiting the characteristics of patients with COPD, the type of mask, and the frequency and duration of mask wearing to minimize the loss of studies related to mask wearing in patients with COPD. In addition, to ensure that we find as many studies as possible, there are no language, year, or country restrictions in this protocol, and data will be collected by searching six databases, including two Chinese-language databases. Based on our preliminary search and screening results and published data, we have identified over 30 studies relating to mask wearing in patients with COPD. Thus, our search strategy, inclusion criteria, and selection of databases can ensure that a systematic review is feasible.” (Discussion, lines 270-280, paragraph 2 page 14-15)

*3. Lines 126-128, Types of interventions may not be comparable relative to the clinical outcome if duration and frequency of wearing facemask are not considered.*

To clarify this point, we have added the following sentences accordingly in the Methods section, it now reads: “2. Different types of facemasks: surgical masks, N95 masks, cloth masks, etc. 3. Characterization of mask wear: duration of mask wear and frequency of mask wear.” (Methods, line 234 paragraph 2 page 12)

*4. It is not clear what mean differences will be collected and summarized. Types of studies include randomized and non-randomized trials, while the proposed statistical analysis will examine mean change at final from mean at baseline. How are these means obtained from randomized and non-randomized trials if their designs are not looking at baseline and final?*

To clarify this point, we have modified our manuscript as follows: “For continuous variables, we will record the baseline value and final value after the intervention for each group, including means and standard deviations. These data will be extracted from tables or text or calculated from other known data when necessary. For trials lacking the mean and SD of the final value, we will use formulas to impute relevant information based on the Cochrane Collaboration Handbook guidelines [25], assuming a correlation coefficient (Corr) of 0.5 [25, 26].”

$${Mean}_{change}={Mean}_{final}-{Mean}_{baseline}$$

$${SD}_{change}=\sqrt{{SD}_{baseline}^{2}+{SD}_{final}^{2}-2\times Corr\times{SD}_{baseline}\times{SD}_{final}}$$

If relevant data are not reported, we will contact the researcher or study sponsor by email to request the missing data, especially for data needed for meta-analysis. When this is not possible, currently available data will be analyzed and discussed as limitations. Heterogeneity across included studies will be assessed using the Q statistic and I-square index. We will use the fixed-effect model for homogeneous studies and the random-effects model for others.” (Methods, line 210-221 paragraph 2 page 11)

*5. Line 209 – 214, if trials don’t provide mean and SD of the final, the proposed formula is not useful.*

As mentioned above, the formula can be used to calculate the final value when the included studies provide the baseline value and the change from baseline value. When final values and baseline values are not given, we will attempt to obtain the data by contacting the researcher or study sponsor.

*6. Please look for help from English editor to the writing.*

This manuscript has been proofread by a native English speaker with scientific background.

We have revised our manuscript accordingly, and hope our manuscript meet the standard for publication in PLOS ONE.

Yours sincerely

Jingchun Fan
