# Supplementary material for: The impact of wearing facemask on COPD patients: A protocol of a systematic review and meta-analysis
Source: PLoS One. 2023 Sep 28;18(9):e0292388. doi: 10.1371/journal.pone.0292388 (PMC10538665; doi:10.1371/journal.pone.0292388)
Supplement: Attachment — Submitted filename: Response to Reviewers-V15.docx [file pone.0292388.s003.docx]

Dr Dong Keon Yon

Academic Editor

PLOS ONE

11 September 2023

Dear Dr Yon

We appreciate the constructive comments made by the reviewers, and our responses are as follows:

Journal Requirements:

*1. Please review your reference list to ensure that it is complete and correct. If you have cited papers that have been retracted, please include the rationale for doing so in the manuscript text, or remove these references and replace them with relevant current references. Any changes to the reference list should be mentioned in the rebuttal letter that accompanies your revised manuscript. If you need to cite a retracted article, indicate the article’s retracted status in the References list and also include a citation and full reference for the retraction notice.*

We confirm that there is no retracted reference cited in our manuscript.

Additional Editor Comments:

Thanks for the positive comment: “*This is an excellent paper.”*

*Please address my minor comment.*

*Ref 18, 19 -> Please updated PRISMA guideline (2015 -> 2020); DOI: https://doi.org/10.54724/lc.2022.e9*

We have modified our manuscript accordingly. It now reads: “The Preferred Reporting Items for Systematic Reviews and Meta-Analysis (PRISMA) will be used to report the systematic review and meta-analysis [21]” (Methods, lines 109-110, paragraph 1 page 6)

21. Lee S W, Koo M J. PRISMA 2020 statement and guidelines for systematic review and meta-analysis articles, and their underlying mathematics: Life Cycle Committee Recommendations. Life Cycle. 2022; 2:e9. https:// doi.org/10.54724/lc.2022.e9

Comments to the Author

*1. Does the manuscript provide a valid rationale for the proposed study, with clearly identified and justified research questions?*

*The research question outlined is expected to address a valid academic problem or topic and contribute to the base of knowledge in the field.*

Reviewer #1: *Yes*

Reviewer #2: *Partly*

Reviewer #4: *Yes*

Thank you.

*2. Is the protocol technically sound and planned in a manner that will lead to a meaningful outcome and allow testing the stated hypotheses?*

*The manuscript should describe the methods in sufficient detail to prevent undisclosed flexibility in the experimental procedure or analysis pipeline, including sufficient outcome-neutral conditions (e.g. necessary controls, absence of floor or ceiling effects) to test the proposed hypotheses and a statistical power analysis where applicable. As there may be aspects of the methodology and analysis which can only be refined once the work is undertaken, authors should outline potential assumptions and explicitly describe what aspects of the proposed analyses, if any, are exploratory.*

Reviewer #1: *Yes*

Reviewer #2: *No*

Reviewer #4: *Yes*

We have modified our manuscript accordingly.

3. *Is the methodology feasible and described in sufficient detail to allow the work to be replicable?*

*Descriptions of methods and materials in the protocol should be reported in sufficient detail for another researcher to reproduce all experiments and analyses. The protocol should describe the appropriate controls, sample size calculations, and replication needed to ensure that the data are robust and reproducible.*

Reviewer #1: *Yes*

Reviewer #2: *Yes*

Reviewer #4: *Yes*

Thank you.

4. *Have the authors described where all data underlying the findings will be made available when the study is complete?*

*The PLOS Data policy requires authors to make all data underlying the findings described in their manuscript fully available without restriction, with rare exception, at the time of publication. The data should be provided as part of the manuscript or its supporting information, or deposited to a public repository. For example, in addition to summary statistics, the data points behind means, medians and variance measures should be available. If there are restrictions on publicly sharing data—e.g. participant privacy or use of data from a third party—those must be specified.*

Reviewer #1: *Yes*

Reviewer #2: *Yes*

Reviewer #4: *No*

We have modified our manuscript in the “Data Availability Statement”, it now reads: “No datasets were generated or analysed during the current study. All relevant data from this study will be made available upon study completion. Upon publication of the study results, a fully dataset will be made publicly available if required by the scientific journal in which the results are published. If the scientific journal does not require a full dataset, a fully dataset will be made available from the corresponding author upon reasonable request.” (Data Availability Statement, lines 306-310, paragraph 2 page 16)

*5. Is the manuscript presented in an intelligible fashion and written in standard English?*

*PLOS ONE does not copyedit accepted manuscripts, so the language in submitted articles must be clear, correct, and unambiguous. Any typographical or grammatical errors should be corrected at revision, so please note any specific errors here.*

Reviewer #1: *Yes*

Reviewer #2: *Yes*

Reviewer #4: *Yes*

Thank you.

6. *Review Comments to the Author*

Reviewer #1: *all comments have been addressed to satisfaction and the article may be accepted if found to be suitable by the editorial board.*

Reviewer #2: *The methodology is still confusing. Study group is mask wearing, while control group is other masks wearing / no mask wearing?*

We apologize for any confusion. To clarify this point, we have modified our manuscript accordingly. It now reads: “The systematic review and meta-analysis will encompass two distinct comparisons: 1. Comparisons involving COPD patients in the mask group and those in the non-mask group. 2. Comparisons within COPD patients who wear different types of masks.” (Methods, lines 130-132, paragraph 2 page 7)

*EtCO_2_ is one of the parameters due for checking. However, it is not routinely done in COPD patients.*

Although ETCO_2_ is not routinely measured in patients with COPD, it is important as an indicator that can be obtained by non-invasive technology to assess CO_2_ levels in mask wearers. In addition, based on our preliminary search and published data, we have identified multiple interventional trials related to masks that have involved ETCO_2_.Therefore, we hypothesize that ETCO_2_ might also be measured in interventional trials of mask wearing in patients with COPD. To clarify this point, we have added the following sentences to the manuscript: “It is worth mentioning that, based on preliminary search and published data, we identified several trials of mask-related interventions involving ETCO_2_ [22-24]. Similarly, the protocol anticipates the importance of ETCO_2_, as an indicator that can be obtained through non-invasive technology, for assessing carbon dioxide levels in mask-wearing COPD patients [25].” (Methods, lines 142-146, paragraph 4 page 7)

22. Epstein D, Korytny A, Isenberg Y, Marcusohn E, Zukermann R, Bishop B, et al. Return to training in the COVID-19 era: The physiological effects of face masks during exercise. Scand J Med Sci Sports. 2021; 31(1):70-5. http://doi:10.1111/sms.13832 PMID: 32969531
23. Lubrano R, Bloise S, Marcellino A, Ciolli CP, Testa A, De Luca E, et al. Effects of N95 Mask Use on Pulmonary Function in Children. J Pediatr. 2021; 237:143-7. http://doi:10.1016/j.jpeds.2021.05.050 PMID: 34043996
24. Bar-On O, Gendler Y, Stafler P, Levine H, Steuer G, Shmueli E, et al. Effects of Wearing Facemasks During Brisk Walks: A COVID-19 Dilemma. J Am Board Fam Med. 2021; 34(4):798-801. http://doi:10.3122/jabfm.2021.04.200559 PMID: 34312270
25. Brooks JP, Layman J, Willis J. Physiologic effects of surgical masking in children versus adults. PeerJ. 2023; 11:e15474. http://doi:10.7717/peerj.15474 PMID: 37342359

Reviewer #4: *All my comments have been addressed. No further comments.*

*The following is my previous comments.*

*This manuscript is a proposal of potential meta-analysis about the impact of wearing facemask on the clinical outcomes of COPD patients. I have below comments.*

*1. It’s not clear about the importance of this topic about the correlation between wearing facemask and the clinical outcomes of COPD patients. If the environment and air quality is good, facemask would be unnecessary for COPD patients.*

*2. It is not clear how many published studies are available for meta-analysis to summarize the correlation between wearing facemask and the clinical outcomes of COPD patients. Authors should have provided such information to confirm the feasibility of the proposed systemic review.*

*3. Lines 126-128, Types of interventions may not be comparable relative to the clinical outcome if duration and frequency of wearing facemask are not considered.*

*4. It is not clear what mean differences will be collected and summarized. Types of studies include randomized and non-randomized trials, while the proposed statistical analysis will examine mean change at final from mean at baseline. How are these means obtained from randomized and non-randomized trials if their designs are not looking at baseline and final?*

*5. Line 209 – 214, if trials don’t provide mean and SD of the final, the proposed formula is not useful.*

*6. Please look for help from English editor to the writing*

We have revised our manuscript accordingly, and hope our manuscript meet the standard for publication in PLOS ONE.

Yours sincerely

Jingchun Fan
